# The Chain Mediating Effects of Parent–Child Conflict and Screen Time on the Relationship Between Parental Phubbing and Problem Behaviors in Preschoolers

**DOI:** 10.3390/bs15020203

**Published:** 2025-02-13

**Authors:** Qiulan Gu, Mei Zhao

**Affiliations:** 1 CAS Key Laboratory of Mental Health, Institute of Psychology, Chinese Academy of Sciences, Beijing 100101, China; lanny_gu@163.com; 2 Department of Psychology, University of Chinese Academy of Sciences, Beijing 101408, China

**Keywords:** parental phubbing behavior, screen exposure, parent–child conflict, child problem behavior

## Abstract

This study aims to investigate the key factors contributing to parental phubbing behaviors (the combination of “phone” and “snubbing”), ultimately reducing behavioral problems and promoting healthy development among preschool children. Parental phubbing refers to the phenomenon where parents neglect their children due to excessive mobile device use during parent–child interactions. A questionnaire was administered to 751 parents of preschool children during 2023 using a convenience sampling methodology. Structural equation modeling was used to investigate the underlying mechanisms among parental phubbing behavior, parent–child conflict, children’s screen exposure duration, and preschool children’s problematic behaviors. The phenomenon of parental phubbing exhibits a medium-high level of prevalence, paralleled by medium-high levels of problematic behaviors in preschool children; Parental phubbing not only directly predicts problematic behaviors in preschool children, but also indirectly influences these behaviors through a mediating chain comprising parent–child conflict and children’s screen exposure duration. However, parental phubbing behavior does not directly predict preschool children’s screen exposure duration; rather, parental phubbing behavior influences children’s screen time through parent–child conflict as a significant mediating factor. The phenomenon of parental phubbing behavior is concerning, suggesting that parents should pay attention to the potential hazards of media use on preschool children, improve their own media literacy, and provide appropriate media guidance to their children while accompanying them at home, so as to jointly promote the comprehensive development of preschool children.

## 1. Introduction

With rapid technological advancement, electronic media’s impact on family relationships and healthy child development has become increasingly prominent. Children’s extended screen time has become a global concern over the past two decades. The American Academy of Pediatrics (AAP) recommends that children aged 2–5 years should not exceed one hour of screen time per day (Council on Communications and Media et al., 2016). The AAP guidelines are internationally recognized and have been widely cited in global research and validated through extensive studies, including those conducted in Asian contexts. Parental phubbing behavior, characterized by excessive smartphone use during parent–child interactions, significantly compromises their ability to meet children’s emotional needs, resulting in diminished quality of parent–child communication (Solecki, 2022). The resultant parental neglect frequently triggers emotional responses of dissatisfaction and anger among children, potentially causing them to exhibit problem behaviors to gain parental attention (Y. J. Wang et al., 2021). The quality of parent–child interaction is inextricably linked to early childhood development. Suboptimal parent–child interaction patterns may precipitate a spectrum of behavioral issues in children, manifesting as emotional regulation disorders, impaired peer interaction capabilities, and delayed language development (Phua et al., 2020; Poulain et al., 2019).

Empirical evidence suggests that up to 25% of preschool children exhibit developmental delays in social–emotional development and behavioral functioning, including emotional self-regulation, verbal communication skills, and both gross and fine motor skills (Browne et al., 2018; Janus & Offord, 2007). The preschool period represents a crucial phase of physical and cognitive development before formal education, and children’s physical and mental health during this stage is vital for their development (Qi et al., 2011). Within Bandura’s social learning theoretical framework (Bandura, 1977), children’s propensity to observe and emulate their parents’ excessive smartphone usage patterns underscores the crucial significance of parental behavioral modeling and instruction during the preschool developmental stage. In their role as primary caregivers, parents influence children and adolescents through both verbal instruction and behavioral demonstration, profoundly shaping the development of their offspring’s linguistic abilities, cognitive development, and self-regulatory mechanisms (Choe et al., 2013). Behavioral difficulties manifested in the preschool years commonly follow stable developmental patterns, yielding substantial impacts on adolescent psychosocial adaptation while potentially persisting through adulthood, affecting behavioral expression. This study aims to investigate the relationship between parental phubbing and preschoolers’ problem behaviors, providing references for improving the quality of family education.

The proliferation of electronic media has given rise to concerning behavioral patterns, particularly excessive electronic device use, which leads to diminished interpersonal interaction and communication (Pathak, 2013). The neologism “phubbing,” derived from the combination of “phone” and “snubbing,” originated from a Macquarie Dictionary initiative in 2012. This term characterizes the act of disregarding present company in favor of mobile phone engagement, alternatively referred to as “phone neglect behavior” (Chotpitayasunondh & Douglas, 2016). When this phenomenon manifests in the parental population, specifically when caregivers’ mobile device usage interferes with parent–child interaction during childrearing, it is designated “parental phubbing”. For preschool children, parents serve dual roles as both caregivers for preschool children and behavioral role models; consequently, children’s electronic screen use patterns frequently mirror their parents’ behaviors (Ji et al., 2015). Empirical studies show that parental phubbing negatively correlates with parent–child interaction quality (Gong et al., 2019; Kildare & Middlemiss, 2017). Excessive mobile device use reduces parental sensitivity to children’s behavioral cues and emotional responsiveness, potentially compromising children’s emotional needs (Vanden Abeele et al., 2020; Kelly & Ocular, 2021). Technology-induced distraction may create feelings of neglect among children, undermining secure attachment development and increasing behavioral problem risks (J. J. Xu & Song, 2024). The parent–child relationship, established through interactions between parents and children, represents an individual’s earliest social relationship, predicated on biological ties and shared living experiences (Y. F. Wang & Feng, 2006). Research conducted by Carr and Dempster reveals that, compared to traditional parent–child activities, parental immersion in mobile devices significantly diminishes the effectiveness of parent–child interactions (Carr & Dempster, 2021). Parental phubbing has evolved into the most prominent form of disruption affecting the quality of parent–child engagement (Knitter & Zemp, 2020). Parental phubbing can lead children to perceive a lack of effective parental responsiveness, potentially resulting in distant parent–child relationships (Przybylski & Weinstein, 2013). Parent–child relationships directly influence children’s externalized behavioral problems, with children in negative parent–child relationships more likely to exhibit aggressive behavior, disciplinary issues, and hyperactivity (Dagan et al., 2021). Empirical evidence indicates that parental smartphone addiction exerts an indirect effect on preschoolers’ social withdrawal tendencies through parent–child conflict as a mediating variable (P. Zhang & Wang, 2025).

In the context of child development research, screen exposure refers to the duration and patterns of engagement with electronic screen devices (such as mobile phones, iPads, gaming consoles, and televisions). Parental smartphone overuse demonstrates a significant association with increased risk of problematic mobile device usage patterns in children (Matarma et al., 2016). Contemporary empirical evidence indicates that prolonged screen exposure is significantly associated with an increased risk of emotional and behavioral difficulties among children across various developmental stages (Kahn et al., 2021; Lin et al., 2015; Sheng et al., 2018). Haidt’s analysis demonstrates a significant association between the widespread adoption of smartphones and social media platforms and the escalating prevalence of mental health concerns among adolescents; the post-2012 cohort, termed “iGen,” confronts mental health challenges of unprecedented magnitude (Haidt, 2024).

Parent–child relationships may influence screen exposure duration, and poorer parent–child relationships and reduced effective communication between parents and children may lead to children spending more time on screens (R. S. Wong et al., 2020). Research indicates that parental smartphone absorption compromises attention allocation to child-directed interactions, leading to reduced quality of parent–child engagement. Deterioration in interpersonal dynamics precipitates child discontentment and subsequent relational conflicts (Kushlev & Dunn, 2019). Conflictual family atmospheres induce negative emotional states in young children, including frustration and anxiety, ultimately diminishing communicative initiatives from offspring (Han & Shek, 2012). Moreover, observational learning of parental device usage patterns may predispose young individuals to seek emotional fulfillment through screen-based interactions (T. Y. Wong et al., 2015).

This study aims to examine the relationship between parental phubbing and behavioral problems in preschool children, investigating the mediating effects of parent–child conflict and children’s screen time exposure, as well as their sequential mediating effect. The following hypotheses are proposed:

**Hypothesis 1** **(H1):** *Parental phubbing behavior positively predicts behavioral problems in preschool children.*

**Hypothesis 2** **(H2):** *The parent–child relationship mediates the relationship between parental phubbing and behavioral problems in preschool children.*

**Hypothesis 3** **(H3):** *Children’s screen time exposure mediates the relationship between parental phubbing and behavioral problems in preschool children.*

**Hypothesis 4** **(H4):** *The parent–child relationship and children’s screen time exposure sequentially mediate the relationship between parental phubbing and behavioral problems in preschool children.*

## 2. Materials and Methods

### 2.1. Participants

Electronic questionnaires were administered using a convenience sampling methodology through the “Wenjuanxing” online survey platform during the period of June to August 2023. Based on the methodological guidelines established by Hair, which stipulate that the minimum sample size should be five times the number of variables, a target sample size of 240 participants was determined for this investigation (Hair, 2010). Distribution channels included WeChat groups and social media networks (WeChat Moments), targeting parents with preschool-aged children (3–7 years). The study participants were predominantly recruited from four regions in China: Guangdong Province, Hunan Province, Shandong Province, and Beijing Municipality. The inclusion criteria were as follows: (1) parents of preschool children aged 3–7 years (7 years old refers to children who have completed 6 years of age but not reached 7 years of age, and do not meet the Ministry of Education’s school enrollment age requirements); (2) clear consciousness and the ability to complete questionnaires independently; and (3) voluntary participation and signed informed consent. The exclusion criteria were as follows: (1) extremely short completion time; (2) regular response patterns; and (3) contradictory information.

This study received approval from the Ethics Committee of the Institute of Psychology, Chinese Academy of Sciences, adhering to ethical principles. The ethics approval number is H23128.

### 2.2. Methods

#### 2.2.1. General Information Questionnaire

The questionnaire was self-designed after reviewing relevant domestic and international literature, divided into child and parent sections. The child section included age, sex, only-child status, and co-residence with parents. The parent section included age, residence, occupation, education level, average monthly family income, and family structure. Given our study population of young children (3–7 years), we collected biological sex (male/female) data rather than gender, as biological sex provides more straightforward assessment opportunities and is less influenced by complex social and cultural factors at this developmental stage.

#### 2.2.2. Parental Phubbing Scale

Adapted from Robert and David’s (2016) Partner Phubbing Scale, which was translated and modified according to Chinese conditions by Ding Qian et al., the single-dimension scale contains 9 items rated on a 5-point scale (1 = strongly disagree, 2 = disagree, 3 = neutral, 4 = agree, 5 = strongly agree), with higher scores indicating more severe parental phubbing behavior. Sample items from the parental phubbing scale include: “I engaged with my mobile device during mealtimes with my child” and “I kept my phone within sight when I was with my child”. The evidence of reliability analysis showed a Cronbach’s alpha of 0.905, indicating good internal consistency. This value aligns well with previously reported reliability in the literature, where Ding reported α = 0.88 in their study with middle school students (Ding et al., 2019). Both values indicate strong internal reliability of this scale in Chinese research contexts.

#### 2.2.3. Parent–Child Relationship Scale

Developed by Driscoll and Pianta (2021), the scale was translated and modified by Chinese scholar Zhang. Based on psychometric analyses which revealed low internal consistency reliability for the dependency dimension, this study employed closeness and conflict dimensions, which demonstrated superior measurement stability (X. Zhang et al., 2008). This study also used these two dimensions, which were scored using 5-point ratings (1 = completely inconsistent, 2 = somewhat inconsistent, 3 = uncertain, 4 = partially consistent, 5 = completely consistent) according to Zu Jing’s method (Zu et al., 2022). Items from the Parent–Child Relationship Scale include the following: “There is a strong affective connection and warmth in my relationship with my child” and “My child demonstrates spontaneous self-disclosure regarding their personal experiences”. Higher total scores indicate poorer parent–child relationships and higher conflict. The evidence of reliability analysis yielded a Cronbach’s alpha of 0.801, demonstrating good internal consistency. This reliability is consistent with established standards, and aligns with prior research such as Liu, who reported a = 0.88 (Liu et al., 2023). These values demonstrate that the scale maintains good internal reliability in Chinese research contexts.

#### 2.2.4. Conners Child Behavior Scale

Developed by Conners in 1969 (Goyette et al., 1978) to assess hyperactivity in children aged 3–17, this scale was revised in 1978 to better reflect preschool problem behaviors. The questionnaire covered six factors: conduct, learning, psychosomatic issues, impulsivity–hyperactivity, anxiety, and hyperactivity, using a four-point scoring system (0 = never, 1 = occasionally, 2 = frequently, 3 = very often). Items from the Conners Child Behavior Scale include “Displayed disrespectful behavior towards adults” and “Exhibited frequent or easily triggered crying episodes”. Scoring was based on X ± 2SD for the normal range (where X represented the mean and SD represented the standard deviation). The evidence of reliability analysis yielded a Cronbach’s alpha of 0.974, indicating excellent internal consistency. This high reliability aligns well with previous research, such as the work of Yang, who reported α = 0.952 in their study on parental phubbing and children’s problem behaviors (Yang, 2022). Both values indicate exceptionally strong internal reliability of this scale in Chinese research contexts.

#### 2.2.5. Children’s Screen Exposure Scale

Modified by Li Minyi et al. based on the international literature (Li & Wang, 2014) and Chinese conditions, the scale measured multimedia use among 3–6 year olds in family settings. This study used items that measured daily screen exposure duration, including television viewing, computer usage, and mobile device interaction, employing an 8-point scoring (1 = “0 min” to 8 = “over 2 h”). The evidence of reliability analysis yielded a Cronbach’s alpha of 0.799, indicating good internal consistency. This reliability aligns closely with Li, who reported α = 0.80 in their research on screen time and children’s physical health (Li et al., 2021). Both values demonstrate that the scale maintains good internal reliability in Chinese research contexts.

### 2.3. Statistical Analysis

Raw data exported from Wenjuanxing was analyzed using Excel, SPSS24.0, and Process 4.2 macro. Analyses included Pearson correlations, independent samples *t*-tests, a one-way ANOVA, and bootstrap mediation effect testing. In calculating the parent–child conflict scores of the Parent–Child Relationship Scale, the closeness dimension was reverse-scored in SPSS and added to the conflict dimension scores. Statistical significance was set at *p* < 0.05.

## 3. Results

### 3.1. Common Method Bias

Harman’s single-factor test was employed to examine common method bias. The results showed that without rotation, 14 factors had eigenvalues greater than 1, with the first factor explaining 28.94% of the variance, which did not reach the critical value of 40%. This indicated no significant common method bias in this study (Podsakoff et al., 2003).

### 3.2. General Characteristics of Participants

In this study, all questionnaires were submitted anonymously. A total of 751 questionnaires were collected, and after screening for those that did not meet the requirements, 612 valid questionnaires remained, resulting in a valid response rate of 81.49%. Among 612 valid questionnaires, male children numbered 264 and female children 348; 328 were only children and 284 were not only children; primary caregivers included 65 fathers, 117 mothers, 408 couples jointly, 18 grandparents, and 4 others; 454 lived with parents while 158 did not; survey respondents included 258 fathers, 338 mothers, 7 grandparents, and 9 others; parents’ highest education levels ranged from illiterate (1) to graduate degree (60), including primary school (10), middle school (43), regular/vocational high school (83), secondary vocational/technical/normal school (36), associate degree (149), and bachelor’s degree (230); monthly average income ranged from CNY ≤ 4000 (68) to CNY ≥ 50,001 (45), with intermediate ranges of CNY 4001–8000 (124), CNY 8001–12,000 (155), CNY 12,001–20,000 (133), CNY 20,001–30,000 (53), and CNY 30,001–50,000 (34); family structures included nuclear families (441), skipped-generation families (29), extended families (128), single-parent families (9), and remarried families (5). The general characteristics of the participants are shown in Table 1.

### 3.3. Demographic Difference Analysis of Main Variables

Independent sample *t*-tests and a one-way ANOVA were conducted to analyze demographic differences in variables, examining whether there were significant statistical differences in four main variables (parental phubbing, parent–child conflict, children’s screen exposure duration, and preschool children’s behavioral problems) across demographic characteristics including the child’s sex, only-child status, monthly family income, and family structure. Regarding the child’s sex, there were no significant differences in parental phubbing (t = −1.94, *p* > 0.05), parent–child conflict (t = 0.303, *p* > 0.05), children’s screen exposure duration (t = 0.748, *p* > 0.05), or behavioral problems in preschool children (t = 0.34, *p* > 0.05); regarding only-child status, there were no significant differences in parental phubbing (t = 0.31, *p* > 0.05), significant differences in parent–child conflict (t = −2.26, *p* < 0.05), no significant differences in children’s screen exposure duration (t = −0.69, *p* > 0.05), and no significant differences in children’s behavioral problems (t = −0.07, *p* > 0.05); regarding family monthly income, there were significant differences in parental phubbing (F = 2.76, *p* < 0.05), parent–child conflict (F = 7.26, *p* < 0.05), children’s screen exposure duration (F = 4.68, *p* < 0.05), and children’s behavioral problems (F = 2.34, *p* < 0.05); regarding family structure, there were significant differences in parental phubbing (F = 3.86, *p* < 0.05) and parent–child conflict (F = 3.55, *p* < 0.05), no significant differences in children’s screen exposure duration (F = 1.27, *p* > 0.05), and significant differences in children’s behavioral problems (F = 4.22, *p* < 0.05). The results are shown in Table 2.

### 3.4. Correlation Analysis of Parent Phubbing, Parent–Child Conflict, Child Screen Exposure Time, and Child Problem Behavior

Table 3 showed the correlation coefficients between parent phubbing, parent–child conflict, child screen exposure time, and child problem behavior. The results indicated that parent phubbing was positively correlated with parent–child conflict (r = 0.380, *p* < 0.01) and child problem behavior (r = 0.443, *p* < 0.01). Parent–child conflict was positively correlated with child screen exposure time (r = 0.141, *p* < 0.01) and child problem behavior (r = 0.594, *p* < 0.01). Child screen exposure time was positively correlated with child problem behavior (r = 0.216, *p* < 0.01). These significant correlations among the four variables warranted further analysis.

### 3.5. Mediation Effect Analysis of Parent–Child Conflict and Child Screen Time in the Relationship Between Parent Phubbing and Preschool Children’s Problem Behaviors

According to the regression analysis validity test results, parent phubbing directly predicted preschool children’s problem behaviors (β = 0.148, *p* < 0.01), indicating a significant direct effect, so hypothesis H1 was supported by the empirical findings. Parent phubbing significantly predicted parent–child conflict (β = 0.175, *p* < 0.01). Parent–child conflict significantly affected child screen exposure time (β = 0.584, *p* < 0.01). Both parent–child conflict (β = 0.562, *p* < 0.01) and child screen exposure time (β = 0.036, *p* < 0.01) significantly affect preschool children’s problem behaviors. See Table 4.

### 3.6. Mediating Effects of Parent–Child Conflict and Child Screen Exposure Time Between Parent Phubbing and Child Problem Behavior

**Figure 1:**
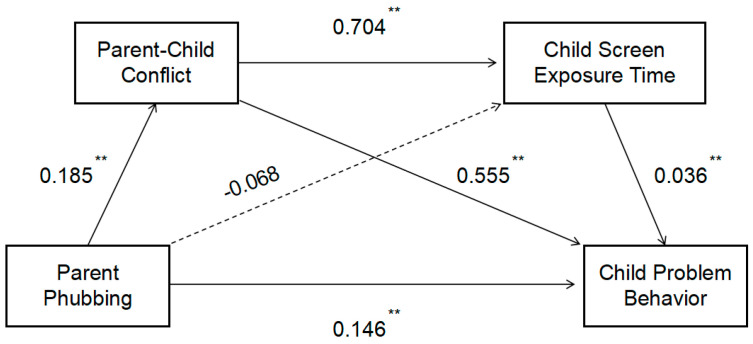
Serial mediation effect of parent−child conflict and child screen exposure time. Note: ** *p* < 0.01.

Given the correlations among parent phubbing, parent–child conflict, child screen exposure time, and child problem behavior, Model 6 in the process was used to examine the mediating effects. A serial mediation model was constructed with child problem behavior as the dependent variable, parent phubbing as the independent variable, and parent–child conflict and child screen exposure time as mediating variables. The path coefficients are shown in Figure 1. The overall regression model was significant (R^2^ = 0.196, F = 148.780, *p* < 0.001). Bootstrap analysis was conducted to further test the mediating effects. The results showed that the indirect effect through parent–child conflict was significant (0.103, 95% CI [0.081, 0.125]), so the research hypothesis H2 was verified; the indirect effect through child screen exposure time was non-significant (−0.002, 95% CI [−0.009, 0.004]), so hypothesis H3 was not supported by the empirical findings; the serial mediation effect through both parent–child conflict and child screen exposure time was significant (0.005, 95% CI [0.001, 0.010]), so hypothesis H4 was supported by the empirical findings. See Table 5 for details.

## 4. Discussion

### 4.1. Parental Phubbing and Problem Behaviors in Preschool Children

This study primarily investigated the mechanism of how parental phubbing influences preschool children’s problem behaviors. Consistent with previous research (Zu et al., 2022), this study demonstrates that parent phubbing positively predicts preschool children’s problem behaviors. Parent phubbing can adversely affect children’s problem behaviors through multiple potential mechanisms, and our findings indicate that parent phubbing influences preschool children’s problem behaviors through parent–child conflict. When parents become excessively engrossed in using smart devices, the quality of parent–child relationships is compromised, subsequently causing distress in children’s emotions and behaviors (Zu et al., 2022). Importantly, children not only reference their parents’ behavior in social interactions, but under parental neglect and alienation, they may also exhibit reduced social behavior, potentially contributing to a lower quality of interpersonal relationships (Ding et al., 2019; C. Y. Xu et al., 2019).

### 4.2. The Chain Mediation Effect of Screen Time and Parent–Child Conflict

Our research found that parent phubbing does not directly predict children’s screen exposure time significantly, but rather indirectly predicts it through the mediation of parent–child conflict. Fang Hongying et al.’s research also suggests that poorer parent–child relationships correlate with more obvious child screen exposure (Fang et al., 2019). This may be because children’s screen exposure time is influenced by parental control, and parents with high literacy tend to value family companionship more. Only when parent phubbing affects the parent–child relationship does the adverse family atmosphere potentially lead to children’s frustration and negative experiences, causing them to turn to electronic screens for temporary satisfaction, which becomes a more critical factor in developing screen dependency.

In summary, when parents frequently engage in phubbing during interactions with their children, this may weaken children’s sense of belonging and their need for parent–child relationships. Children who consistently observe their parents’ phubbing behavior often tend to invest their emotions in electronic screens to fill their psychological void (T. Y. Wong et al., 2015). Increased parent phubbing makes children feel more emotionally neglected in communication with their parents. At this stage, children have not yet developed good emotional regulation abilities, so they are more likely to express their dissatisfaction through behavioral problems to gain parental attention (Y. J. Wang et al., 2021). Furthermore, frequent use of electronic media triggers and reinforces problem behaviors in preschool children.

Therefore, parent phubbing not only affects the quality of interaction among family members, but also has profound impacts on children’s development. Parents need to recognize how their behavior influences their children and strive to improve the quality of interactions with their children, reduce time spent on electronic devices, spend more time with their children, pay attention to their growth and emotional needs, promote healthier family interaction patterns, and create a more supportive and caring environment for their children’s development.

### 4.3. Theoretical Contributions and Practical Implications

This research advances theoretical understanding through a comprehensive family-oriented perspective, which views parental phubbing not as an isolated parental behavior, but as part of broader family dynamics. Ecological systems theory maintains that during individual development, there exists a reciprocal interaction between environmental contexts and individual characteristics. Optimal family functioning serves as a protective mechanism that enhances children’s psychological resilience, thereby reducing the manifestation of maladaptive behavioral patterns (Zeng & Li, 2003). As the primary microsystem in individual development, the quality of family functioning directly influences children’s psychosocial developmental trajectory. Specifically, efficient family interaction patterns and emotional support networks among family members can satisfy children’s needs for self-worth and belongingness, thereby facilitating the formation of prosocial behaviors (J. Wang et al., 2016). Conversely, the deterioration of family functioning may lead to children’s maladaptive responses and high-risk behavioral patterns (Abar et al., 2015). Rather than attributing blame to parents, our family-oriented lens emphasizes the interactive nature of parent–child relationships and the contextual factors that influence digital device usage within families. By examining bidirectional relationships among parental phubbing behavior, parent–child conflict, and preschool children’s problem behaviors, we highlight the importance of understanding family interactions as an interconnected system and suggest that effective interventions should focus on psychoeducation and family-based support rather than parental criticism. This study addresses a critical gap in both the domestic and international literature by examining these relationships specifically in families with preschool children, where previous research has primarily focused on phubbing’s impact on romantic relationships, corporate settings, workplaces, and educational contexts, particularly among adolescents and college students. The findings provide valuable implications for family-centered interventions, suggesting that mental health practitioners should work collaboratively with both parents and children to develop balanced approaches to technology use while strengthening family bonds. Family intervention strategies for parental phubbing behavior are as follows: first, establish structured time management through scheduled parent–child interactions and device usage boundaries; optimize the environment by creating device-free zones and interactive spaces; enhance interaction quality through diverse activities and effective communication methods; strengthen parental self-management by developing awareness and controlling smartphone usage. These comprehensive strategies require consistent family commitment to reduce the negative impacts of parental phubbing on children.

### 4.4. Research Limitations and Perspectives

Phubbing represents a persistent behavioral state. This study employed a cross-sectional quantitative approach to examine the relationships among parental phubbing, parent–child conflict, children’s screen time, and preschool children’s problem behaviors at a single time point. Such a methodology presents limitations in establishing causal relationships between variables. For future research, longitudinal studies are recommended to investigate whether parental phubbing influences preschool children’s subsequent emotional development, interpersonal relationships, and academic performance, thereby allowing us to understand the developmental trends among these variables. Secondly, due to the employment of a convenience sampling methodology and a relatively limited sample size, the sample may not fully represent the target population, which could affect the generalizability of our findings to broader populations. Thirdly, certain demographic groups might be over-represented or under-represented in our sample. Future research should consider employing more systematic sampling methods to validate and extend these findings across diverse populations and contexts. Moreover, considering that the preschool period constitutes a critical phase in children’s cognitive development and represents a dynamic developmental process, age-specific variations may demonstrate heightened sensitivity to familial environmental influence. Furthermore, the Parental Phubbing Scale utilized in this study, being adapted from international instruments, may not adequately reflect Chinese sociocultural characteristics. The current domestic literature lacks validated instruments for measuring parental phubbing behavior within the Chinese context. Future research should focus on developing culturally appropriate, indigenous assessment tools specifically tailored to the Chinese sociocultural environment. Additionally, subsequent studies should conduct more detailed analyses examining the correlations between phubbing behavior and various occupational categories, including, but not limited to, civil servants, self-employed professionals, and law enforcement officers. Finally, as this study was conducted in mainland China with a predominantly Han Chinese population (95%), racial and ethnic factors were not considered as confounding variables. While this demographic homogeneity limits the generalizability of our findings, future cross-cultural studies should incorporate racial and ethnic variables to enhance result generalizability.

## 5. Conclusions

The preschool period is a critical stage for shaping healthy behaviors and cultivating positive habits. Through questionnaire surveys, this study investigated the relationship between parental phubbing and problem behaviors in preschool children, as well as the mediating effects of parent–child conflict and children’s screen time exposure. The findings revealed that parental phubbing directly predicts problem behaviors in preschool children, with parent–child conflict and children’s screen time exposure serving as chain mediators between parental phubbing and children’s problem behaviors. This research not only enriches the literature on emerging variables, but also broadens the domestic research perspective on the relationship between parental phubbing and preschool children’s problem behaviors. It deepens the understanding of parental phubbing behavior, raises parental awareness, and highlights the importance of regulated smartphone use among parents. The findings contribute to existing educational theories and provide valuable references for children’s mental health guidance.

## Figures and Tables

**Table 1:** Demographic characteristics of participants (N = 612).

Variable	Category	N	Percentage (%)
Child’s Sex	Male	264	43.1
	Female	348	56.9
Only Child	Yes	328	53.6
	No	284	46.4
Primary Caregiver	Father	65	10.6
	Mother	117	19.1
	Both Parents	408	66.7
	Grandparents	18	2.9
	Others	4	0.7
Living with Parents	Yes	454	74.2
	No	158	25.8
Relationship to Child	Father	258	42.2
	Mother	338	55.2
	Grandparents	7	1.1
	Others	9	1.5
Parents’ Highest Education Level	Illiterate/Minimal Literacy	1	0.2
	Primary School	10	1.6
	Middle School	43	7.0
	Regular/Vocational High School	83	13.6
	Secondary Vocational/Technical School	36	5.9
	Associate Degree	149	24.3
	Bachelor’s Degree	230	37.6
	Graduate Degree	60	9.8
Monthly Average Income (CNY)	≤4000	68	11.1
	4001–8000	124	20.3
	8001–12,000	155	25.3
	12,001–20,000	133	21.7
	20,001–30,000	53	8.7
	30,001–50,000	34	5.6
	≥50,001	45	7.4
Family Structure	Nuclear Family	441	72.1
	Skipped-generation Family	29	4.7
	Extended Family	128	20.9
	Single-parent Family	9	1.5
	Remarried Family	5	0.8

**Table 2:** Demographic characteristics of participants continued (N = 612).

Item	Sample Size (N)	Parent Phubbing	Parent–Child Conflict	Child Screen Exposure Time	Child Problem Behavior
		(M ± SD)	(M ± SD)	(M ± SD)	(M ± SD)
Child Sex					
Male	264	3.19 ± 1.02	2.48 ± 0.49	3.27 ± 2.59	0.79 ± 0.59
Female	348	3.35 ± 1.00	2.47 ± 0.50	3.12 ± 2.02	0.77 ± 0.57
t-value		−1.94	0.30	0.748	0.34
*p*		0.05	0.76	0.455	0.74
Only child					
YES	328	3.29 ± 1.02	2.43 ± 0.51	3.13 ± 2.40	0.77 ± 0.59
NO	284	3.27 ± 1.02	2.52 ± 0.47	3.26 ± 2.15	0.78 ± 0.56
t-value		0.31	−2.26	−0.69	−0.07
*p*		0.76	0.02 *	0.488	0.94
Monthly income (CNY)					
≤4000	68	3.49 ± 1.03	2.74 ± 0.41	3.89 ± 2.38	0.91 ± 0.61
4001–8000	124	3.17 ± 0.96	2.58 ± 0.45	3.64 ± 2.54	0.82 ± 0.55
8001–12,000	155	3.48 ± 1.03	2.45 ± 0.52	3.21 ± 2.41	0.79 ± 0.60
12,001–20,000	133	3.25 ± 1.11	2.41 ± 0.47	2.89 ± 1.75	0.78 ± 0.56
20,001–30,000	53	3.10 ± 0.90	2.30 ± 0.45	2.39 ± 1.71	0.59 ± 0.44
30,001–50,000	34	2.99 ± 0.94	2.27 ± 0.54	2.19 ± 1.27	0.60 ± 0.48
≥50,001	45	3.10 ± 0.86	2.40 ± 0.5	3.39 ± 2.89	0.72 ± 0.68
F-value		2.76	7.26	4.68	2.34
*p*		0.01 *	0.00 **	0.00 **	0.03 *
Family structure					
Nuclear family	441	3.36 ± 1.03	2.49 ± 0.49	3.23 ± 2.32	0.8 ± 0.59
Grandparent family	29	3.41 ± 1.03	2.72 ± 0.37	3.53 ± 2.25	1.07 ± 0.67
Extended family	128	2.98 ± 0.87	2.38 ± 0.49	2.9 ± 2.17	0.63 ± 0.48
Single-parent family	9	3.12 ± 1.06	2.37 ± 0.65	4.25 ± 2.69	0.79 ± 0.61
Remarried family	5	3.31 ± 1.66	2.69 ± 0.4	2.62 ± 1.11	0.75 ± 0.48
F-value		3.86	3.55	1.274	4.22
*p*		0.00 **	0.00 **	0.279	0.00 **

Note: M = mean; SD = standard deviation; * *p* < 0.05; ** *p* < 0.01.

**Table 3:** Correlation analysis of variables (N = 612).

	1	2	3	4
Parent Phubbing	1			
Parent–Child Conflict	0.380 **	1		
Child Screen Exposure Time	0.028	0.141 **	1	
Child Problem Behavior	0.443 **	0.594 **	0.216 **	1

Note: ** *p* < 0.01; parent–child relationship is reverse-scored, with higher scores indicating poorer parent–child relationship and higher parent–child conflict.

**Table 4:** Analysis of demographic differences across variables.

Variables	DV: Parent–Child Conflict			DV: Child Screen Time			DV: Child Problem Behavior		
	β	SE	*p*	β	SE	*p*	β	SE	*p*
Child Sex	−0.04	0.037	0.275	−0.099	0.185	0.595	−0.027	0.036	0.451
Child Age	0.033	0.018	0.06	−0.05	0.089	0.57	−0.009	0.017	0.618
Income Level	−0.054	0.011	<0.01	−0.173	0.057	<0.01	0.011	0.011	0.317
Parent Phubbing	0.175	0.018	<0.01	−0.065	0.098	0.508	0.148	0.019	<0.01
Parent–Child Conflict				0.584	0.205	<0.01	0.562	0.04	<0.01
Child Screen Time							0.036	0.008	<0.01
R^2^	0.185			0.036			0.429		
F	34.421 ***			4.570 ***			75.883 ***		

Note. *** *p* < 0.001.

**Table 5:** Serial mediation effect test of parent–child conflict and child screen exposure time (N = 612).

Mediation Effects	Effect Size	SE	95% CI		Relative Effect
			Lower	Upper	
Total effect of phubbing on child problem behavior	0.251	0.021	0.210	0.291	/
Direct effect of phubbing on child problem behavior	0.146	0.019	0.109	0.183	58.17%
Indirect effect of phubbing on child problem behavior	0.105	0.012	0.082	0.129	41.83%
Phubbing → parent–child conflict → problem behavior	0.103	0.011	0.081	0.125	41.04%
Phubbing → screen exposure → problem behavior	−0.002	0.003	−0.009	0.004	−0.01%
Phubbing → parent–child conflict → screen exposure → problem behavior	0.005	0.002	0.001	0.010	0.02%

## Data Availability

The datasets used and/or analyzed during the current study are available from the corresponding author upon reasonable request.
